# Expression of Concern on “*Crocus sativus* L. (Saffron) Stigma Aqueous Extract Induces Apoptosis in Alveolar Human Lung Cancer Cells through Caspase-Dependent Pathways Activation”

**DOI:** 10.1155/2019/2726761

**Published:** 2019-10-22

**Authors:** BioMed Research International

*BioMed Research International* would like to express concern with the article titled “*Crocus sativus* L. (Saffron) Stigma Aqueous Extract Induces Apoptosis in Alveolar Human Lung Cancer Cells through Caspase-Dependent Pathways Activation” published in *BioMed Research International* in 2013 [1] after concerns were raised about figure duplication.

As noted on PubPeer, there are indications of duplication of figure elements in each panel of Figure 2 and in Figure 7B. The authors said they were unable to provide the original high-resolution underlying images for Figures 2 and 7, any replicates, and the individual data points behind Table 1 and Figures 1 and 4 due to a laptop failure, and they offered to replicate the work. The Editorial Board agreed to allow the authors to attempt to replicate this work under the supervision of their institution. This notice may be updated or replaced based on the outcome of the replication attempt and institutional investigation.
